# 

**Published:** 1885-10-20

**Authors:** 


☞ **Notice.**--- Subscribers in arrears are requested to remit their dues at once. Please take due notice and govern yourselves accordingly.

DR. S. H. GRAY, of Barnesville, Ga., died August 31, last.

DR. F. A. STANFORD, of Columbus, Ga., died in September last.

DR. ROBERT CAMPBELL, an esteemed and prominent physician of Augusta, Ga., died September 22, 1885.

DR. THOMAS WAUGH, of Chicago, was shot in September last by a jealous husband.

DR P. O. HOOPER, of Little Rock, Ark., has been appointed Superintendent of the Insane Asylum of that State.

DR F. D. CUNNINGHAM, formerly Professor of Anatomy in the Medical College of Virginia, died on the 9th of September last.

SOUTHERN MEDICAL COLLEGE.—The Introductory Address in this School was delivered on Tuesday, October 6, by A. G. Hobbs, M. D., Professor of the Diseases of the Eye, Ear, and Throat. The address was admirable, and delivered to an increased number of matriculates.

SMALL POX has been prevailing with considerable fatality in Canada, due to the fact that the people are prejudiced against vaccination. The authorities have been compelled to resort to compulsory vaccination in the face of opposition amounting to riots and resistance in the civilized community of Montreal.

*RECEIPTED.*

1885—Drs. T. A. Gibson, M. E. Demaset, Moses Yarbrough, James T. Stacey, Leonard Powell, John Pullam, James Meyers, P. P. Simon, Robert Meigs; M. L Mahaffy, to May 1.

1883—L. B. Bouchell, Thos. W. Walton.

1884—Wm. Simpson, M. M. Snider, I. L. Morgan.

